# Interferon-Mediated Response to Human Metapneumovirus Infection

**DOI:** 10.3390/v10090505

**Published:** 2018-09-18

**Authors:** Ifeanyi K. Uche, Antonieta Guerrero-Plata

**Affiliations:** 1 Department of Pathobiological Sciences, Louisiana State University, Baton Rouge, LA 70803, USA; iuche1@lsu.edu; 2 Center for Experimental Infectious Disease Research, Louisiana State University, Baton Rouge, LA 70803, USA

**Keywords:** human metapneumovirus, HMPV, interferon, IFN, pneumovirus, respiratory

## Abstract

Human metapneumovirus (HMPV) is one of the leading causes of respiratory diseases in infants and children worldwide. Although this pathogen infects mainly young children, elderly and immunocompromised people can be also seriously affected. To date, there is no commercial vaccine available against it. Upon HMPV infection, the host innate arm of defense produces interferons (IFNs), which are critical for limiting HMPV replication. In this review, we offer an updated landscape of the HMPV mediated-IFN response in different models as well as some of the defense tactics employed by the virus to circumvent IFN response.

## 1. Introduction

### 1.1. Structural Characteristics of HMPV

**Figure 1:**
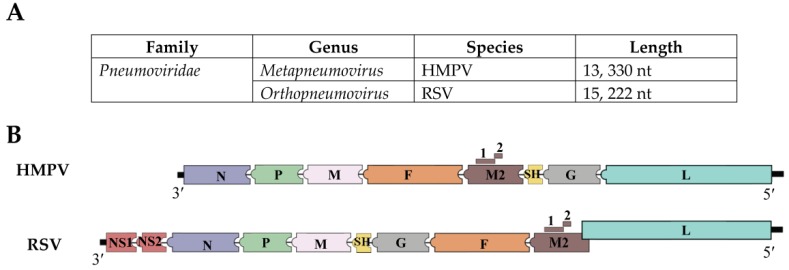
HMPV and RSV genomic configuration and the proteins they encode. (**A**) HMPV and RSV are negative sense single stranded RNA viruses and are members of the family *Pneumoviridae*. HMPV belongs to the genus *Metapneumovirus*, while RSV belongs to *Orthopneumovirus* genus; (**B**) Both viruses differ in the organization of the genomic proteins. Besides, the M2 and L proteins of RSV overlap by 68 nucleotides [16]. Another important difference between the viruses is that RSV genome encodes additional two proteins, non-structural proteins NS1 and NS2.

Human metapneumovirus (HMPV) is a clinically relevant single stranded RNA virus that belongs to the genus *Metapneumovirus* in the new virus family *Pneumoviridae* [1,2,3]. HMPV genomic organization resembles that of the respiratory syncytial virus (RSV) with the exceptions of lacking the non-structural proteins (NS1 and NS2) and of having a slightly different gene order (Figure 1). The HMPV genome, which is constituted of approximately 13,330 nucleotides, encodes 9 proteins represented in the order 3′-N-P-M-F-M2(1/2)-SH-G-L-5′, and includes the nucleoprotein (N), phosphoprotein (P), fusion (F), small hydrophobic (SH), attachment (G), polymerase (L), and matrix proteins (M1 and M2), for which M2 has two overlapping open reading frames (ORF) in the M2 mRNA that generates M2-1 and M2-2 proteins. [4,5,6] (Figure 1). Functional characterization of the HMPV viral proteins indicates that the virus fuses with the host cell membrane using the F protein [7]. Unlike the G protein, with its high genetic diversity, the F protein is considered the most conserved protein among other HMPV isolates [8]. The G protein contributes to the attachment of the virus to the host cell membrane, but is dispensable for virus replication [9]. The small hydrophobic (SH) protein has been reported to modulate membrane fusion promotion [10], however its functional role in viral infection is still unknown. The phosphoprotein (P), nucleocapsid (N), and viral polymerase (L) are essential proteins for viral RNA synthesis [6,10,11]. M2-1 protein appears to be necessary for HMPV replication in vivo [6,12]. HMPV has been classified into two main groups, i.e., A and B, and further subdivided into A1, A2, B1, and B2, based on genetic analysis [8,13,14,15].

### 1.2. Epidimiological Aspects of HMPV

Although discovered in 2001, HMPV has been present in the human population for at least 50 years before it was identified in nasopharyngeal aspirates by Osterhaus’ group in the Netherlands [17]. This virus is known to be one of the most significant causes of acute respiratory tract infection (ARTI) in humans [18]. Since its discovery, cases of HMPV infections have been isolated on all continents [19]. Like human RSV, HMPV has a seasonal distribution and causes periodic epidemics which usually peak during the winter and spring [20,21]. The prevalence of HMPV infections is higher in infants and children (less than 5 years old) than in adults [18,19]. By the age of 5, almost all children have been exposed to HMPV [17,22,23]. Moreover, epidemiological and clinical studies show that HMPV is a major causative agent of severe respiratory infection associated with 5–15% of pediatric hospitalizations [19,24,25]. Data from two different studies indicate that HMPV was detected in nasal-wash samples of 12% of all lower respiratory infection (LRI) cases in a cohort of more than 2000 previously healthy outpatient infants and children [26], as well as in the respiratory aspirates of 12 (6%) of the 208 children hospitalized for ARTI [24]. Based on studies comprising young and older adults, HMPV disease can also occur in adults of all ages [19], although increased severity of the disease is observed in the elderly and immunocompromised individuals [19,27]. Furthermore, stem-cell transplant recipients and people with chronic obstructive pulmonary disease (COPD) have been reported to have severe HMPV infection [28,29].

HMPV is transmitted via direct or close contact with infected individual secretions, which may involve droplets or large particle aerosols [30,31,32]. The incubation period varies among individuals but it is estimated to be between 3–6 days [33,34]. Typical HMPV syndrome includes fever, acute wheezing, and in severe cases, bronchiolitis and pneumonia [26,35,36]. Purulent cough, sore throat, otitis media, bronchitis, and asthma exacerbation are other symptoms that have been reported [34]. Like RSV, factors like age, prematurity, gender, severe lung disease, immunodeficiency, and severe neurological disabilities increase the risk of severe HMPV infection [37,38,39,40,41,42,43,44]. Despite the disease prevalence, there is no available commercial vaccine or treatment against HMPV. However, ribavirin, together with immunoglobulins, has been used to treat infected patients [45]. Other supportive treatments for the infection, which involve the use of fusion inhibitors and small interfering ribonucleic acids, have been reported in several in vitro and animal studies [46,47].

## 2. Interferon Response

### 2.1. Types of Interferons (IFNs)

The production of interferon (IFN) is one important way by which a host immune system responds to invading pathogens such as viruses, bacteria, and parasites [48]. Interferons induced by viral infection constitute the first line of the innate antiviral immune defense. There are three types of interferons: type I IFN, a multi-gene cytokine family that includes several subtypes (α, β, ε, δ, κ, τ, ω, and ζ) [49,50], although the best characterized are IFN-α and IFN-β. In addition, human IFN-α is further classified into 13 subtypes, all in chromosome 9 [51]. Most cell types are able to produce IFN-β, whereas IFN-α is primarily released by plasmacytoid dendritic cells [52]. Type II IFN consists only of the gamma form (IFN-γ), which is produced by NK cells, NKT cells, CD4+ T cells (Th1), CD8+ cytotoxic T lymphocytes, and B cells [53]. Type III IFNs are the newly discovered member of the interferon family which includes 4 isoforms of IFN lambda (IFN-λ1-4) [54,55] and is produced by epithelial cells and plasmacytoid dendritic cells [56,57].

### 2.2. Activation of the IFN Response

**Figure 2:**
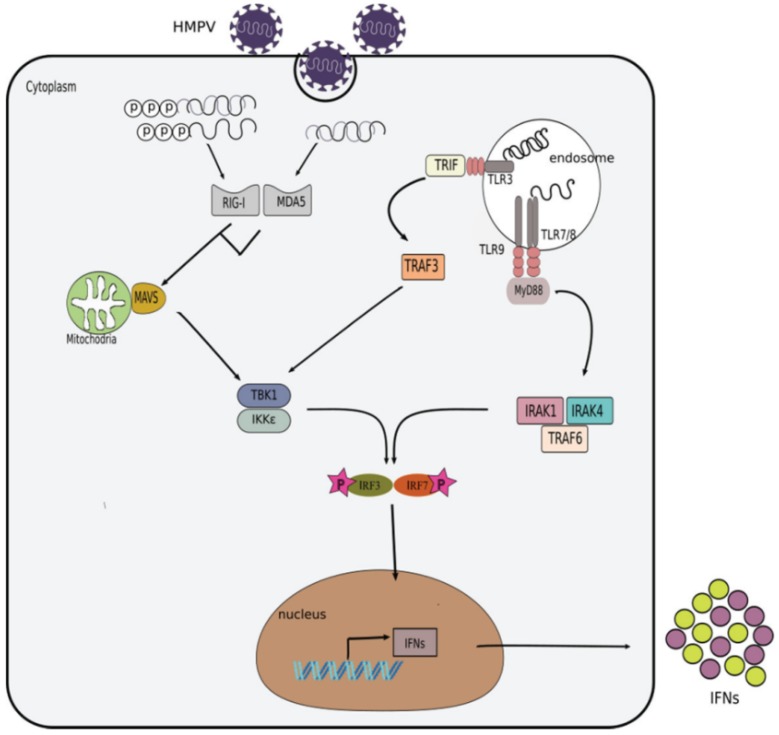
Induction of the interferon response by HMPV. During HMPV infection, its viral PAMPs are detected by toll-like receptors (TLRs) in the endosome and RIG-I-like receptors (RLRs) in the cytoplasm. Activation of TLR7/8 by ssRNA results in the recruitment of the adaptor molecule myeloid differentiation primary response protein 88 (MyD88). MyD88 further recruits and activates the interleukin 1 receptor associated kinases (IRAK1 and IRAK4) and the tumor necrosis factor receptor associated factor 6 (TRAF6) which in turn mediate the phosphorylation and activation of IRF7. Translocation of IRF7 into the nucleus triggers the expression of IFNs. In addition, dsRNA can be detected by TLR3 also in the endosome, which leads to the activation of TRAF3 and subsequent activation of the tank binding kinase-1 (TBK1)/I kappa B kinase epsilon (IKKɛ) kinases that eventually induce the phosphorylation of interferon regulatory factor 3 (IRF3) and IRF7. On the other hand, retinoic acid-inducible gene I (RIG-I) or melanoma differentiation associated factor 5 (MDA5) can be activated in the cytoplasm by 5′ppp-dsRNA, 5′ppp-ssRNA or long dsRNA, respectively. Activation of RIG-I or MDA5 subsequently activates the mitochondrial antiviral signaling protein (MAVS). MAVS recruits additional adaptor proteins that trigger the activation of TBK1/IKKε kinases. TBK1/IKKε phosphorylate IRF3/7 which then translocate to the nucleus to induce the expression of interferons.

Cells in the respiratory tract, such as epithelial cells, phagocytes, dendritic cells, innate lymphoid cells (ILCs), natural killer (NK) cells, among others, use receptors called pattern recognition receptors (PRRs) to recognize pathogen associated molecular patterns (PAMP) present in viral pathogens. Apart from cell surfaces, these receptors (PRRs) are also present in different cellular compartments such as endosome and cytosol. Those PRRs present in the endosomes are the toll-like receptors (TLRs), which can recognize different viral products, including the following: TLR3 (double stranded RNA, dsRNA), TLR7, TLR8 (single stranded RNA, ssRNA), and TLR9 (unmethylated DNA) [58,59]. In the cytoplasm, PRRs include RIG-I like receptors (RLRs) such as RIG-I (retinoic acid–inducible gene I) and melanoma differentiation-associated 5 (MDA5), which sense viral genomic dsRNA, or dsRNA produced as viral intermediates during the virus replicative cycle. Typically, RLRs recognize the following different molecules of dsRNA: MDA5 recognizes dsRNA that is over 2000 nts in length while RIG-I recognizes shorter (<400 nt) structures of dsRNA or ssRNA with 5′ triphosphorylated (5′ppp) modification [60,61,62,63,64]. The detection of viral PAMPs by cellular PRRs leads to the activation of different signaling cascades that result in the production of IFNs by the infected cells, as described in (Figure 2).

### 2.3. Regulation of the IFN Response

Most cells respond early to viral infection by producing IFNs to induce an antiviral response. Depending on the cell type and viral infection, detection of viral PAMPs by cellular PRRs leads to the activation of several down-stream cell signaling cascades that eventually results in the induction of several IFN-inducible genes (such as IFN-stimulated genes; ISG), which may include protein-coding and non-coding microRNA (miRNA) to establish an antiviral state [65]. The creation of an anti-viral environment is accompanied by secretion of IFNs that interfere with the replication of the virus. Hence, IFN, ISGs, and miRNA are part of a cellular innate immunity that responds to virus infection. miRNAs have been of research interest since their discovery in 1993 [66]. They are non-coding RNAs of approximately 22 nucleotides in length that are involved in the post-transcriptional regulation of gene expression [67,68,69]. miRNAs play important roles in diverse biological processes such as cellular differentiation and development, fat metabolism, pathogenesis of various diseases, and immune response [70,71]. The regulatory capabilities of miRNAs may be beneficial or otherwise, depending on the mRNA target they bind to. Thus, miRNA regulating the expression of interferon genes may directly or indirectly inhibit or stimulate viral replication. For instance, expression of miR-22 blocks interferon production by directly downregulating the high mobility group box-1 (HMGB1) and interferon regulatory factor 5 (IRF-5) that eventually inhibits NF-κB and interferon regulatory factor 3 (IRF3) activation [72]. On the other hand, miR-155 enhances innate antiviral immunity during Hepatitis B virus (HBV) infection by targeting the suppressor of cytokine signaling 1(SOCS1) leading to activation of the JAK/STAT signaling pathway [73]. Work from our laboratory and other groups have demonstrated that HMPV infection induces different types of miRNAs in different cell types [74,75]. Although several studies have implicated miRNAs in different lung diseases [76], future work is needed to determine the potential role of miRNAs in the IFN response upon HMPV infection.

Numerous ISGs have been shown to be involved in the regulation of immune responses. Some have been reported to interfere with the replication of different viruses [77,78,79,80], while others have not been characterized in regards to that effect. Using an overexpression screening assay, Schoggins et al. [80] have catalogued several ISGs that might have an inhibitory or enhancing effect on different DNA and RNA viruses. Their results show CD9, HPSE, P2BY6, and interferon induced transmembrane protein 3 (IFITM3) as the top hits of ISGs for HMPV inhibition. In line with that, recent studies show that the expression of IFITM3 dysregulates HMPV F protein mediated cell to cell fusion and inhibits HMPV infection in vitro [81]. They also found that over expression of IFITM1 and IFITM2 can impede HMPV infection [81]. On the other hand, it has been previously demonstrated that the expression of the 3 IFITM proteins mentioned above, effectively curtail RSV infection in vitro. However, unlike in HMPV, the IFITM proteins only interfere with RSV entry [82]. The restriction effect of IFITM3 against RSV has also be demonstrated in a knockout mouse model [83]. Additionally, the inhibitory effects of viperin and ISG15 against HRSV have been described previously [84,85]. Overall, there is limited information on the antiviral activity of IFN-induced proteins against HMPV replication.

## 3. Induction of Interferon by HMPV Infection

Several studies have shown that infection with different strains of HMPV induce a strong immune response characterized by production of interferons in vitro [57,86,87,88,89] or in vivo [57,88,90,91,92,93] (Table 1). However, this response is contingent on viral replication as IFN induction is abolished in UV-inactivated HMPV infected cells [57,94,95] and in vivo [90].

### 3.1. In Vivo (Clinical Studies)

The study of the IFN response in HMPV-infected individuals is very limited. However, studies with nasopharyngeal aspirates from children infected with HMPV indicate that IFN-β is induced by HMPV A group, while IFN-λ was induced by both A and B groups. However, IFN-γ was not detected in those samples [98]. Similarly, in a study by Melendi et al. [99], a reduced IFN-γ/IL-4 ratio was detected in nasal secretions from infants positive for HMPV [99]. However, in a different study, it was shown that IFN-γ was found in nasal airway secretions, but unlike in RSV, that induction was significantly reduced in premature infants infected with HMPV [100].

### 3.2. Models In Vitro (Epithelial and Immune Cells)

Airway epithelial cells are the primary target for HMPV replication [96,101]. Similar to RSV, human alveolar epithelial (A549) cells are also susceptible to HMPV infection [96,102]. Experimental evidence indicates that HMPV infection of A549 cells induces the expression of RIG-I and MDA5, but only uses the RIG-I signaling pathway to initiate the expression of IFN-β [87]. Further studies demonstrate that infection of A549 cells with HMPV induces the production of both IFN-α and IFN–β in a time-dependent manner, but higher amounts of IFN-β than IFN-α was produced in this model [96]. More recently, we observed that HMPV is also able to induce the expression of IFN-λ1-4 in A549 cells, and in higher levels than those of IFN-β [57].

The human and mouse dendritic cells (DCs) are another in vitro model that has been used for the study of the IFN response to HMPV infection. DCs are among the first immune cells to respond during viral invasion [103]. Lung DCs respond to viral infection by secreting type I IFN, which may result from the recognition of viral nucleic acids by TLRs or other PPRs together with the activation of some signaling pathways that lead to the inhibition of viral replication and destruction of the viral genome. In that regard, our laboratory has shown that HMPV-induced signaling activates type I and type III IFN responses in human monocyte-derived dendritic cells (moDC) and murine bone marrow derived dendritic cells (BMDC) through MDA5 [88]. Our discovery was in agreement with previous reported evidence that HMPV differentially activate human DCs and may utilize different tools to modulate a host immune response [104]. However, the distinct PRRs activation by HMPV in epithelial cells and human or mouse DCs, to induce IFN response, can be attributable to the inherent differences of the experimental cell type used. TLR4 has also been implicated as an essential player in the activation of type I IFN in moDC after HMPV infection [105]. Moreover, data in peripheral blood mononuclear cells (PBMCs) infected with HMPV indicate that this virus induces a weaker IFN-γ response when compared to RSV [106].

### 3.3. Experimental Animal Models

Similar to the aforementioned in vitro findings, studies in vivo demonstrate that HMPV is a more potent inducer of IFN-α than RSV [57,91]. We also reconfirmed that HMPV not only induces IFN-α, but also induces IFN-γ and IFN-λ in higher levels than those by RSV [57,92]. It has been claimed that the presence of the NS1 and NS2 proteins found in RSV but not in HMPV, might be a key player for this phenomenon [107,108]. The interaction of type I IFNs with the IFN-α/β receptor (IFNAR) is essential for the activation of the host protective anti-viral response against HMPV [109]. In a model of IFNAR deficient mice infected with HMPV, the lack of IFNAR resulted in an increased viral load, reduced weight loss, and less pulmonary inflammation, but significantly impaired HMPV-specific CD8+ T cell response [109]. Hence, IFNAR signaling appears to play an important role in HMPV lung clearance and disease.

Finally, the role of aging on the IFN response after HMPV infection, has been largely understudied. However, data in BALB/c mice indicate that aged animals have an impaired capacity to produce IFN-γ that the younger counterparts [110]. Thus, it appears that premature or elderly individuals have a limited capacity to mount an IFN response to HMPV. However, future studies are needed to understand the mechanisms underlying those effects.

## 4. Inhibition of IFN Response by HMPV

**Figure 3:**
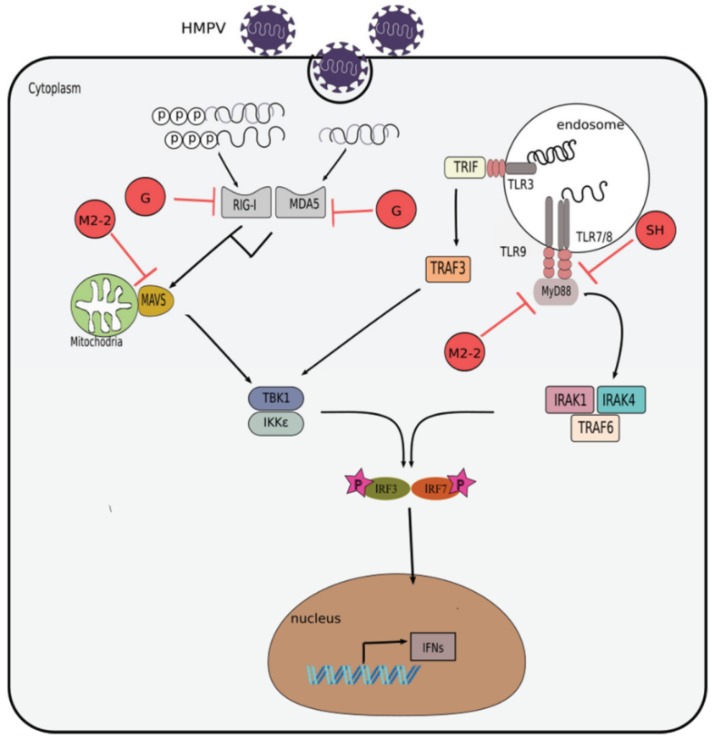
Inhibition of interferon response by HMPV. Evasion mechanisms used by HMPV include: impairment of RIG-I and MDA5 signaling by its G protein; attenuation of MAV or MyD88 signaling by its M2-2 protein; and obstruction of TLR7/MyD88 signaling by its SH protein.

The use of the reverse genetics technique has allowed the study of HMPV proteins individually. Several studies have demonstrated how HMPV uses its proteins for viral replication and to modulate host cell responses, including the IFN-driven antiviral response. Table 2 and Figure 3 summarizes the effect that the attachment (G), matrix (M2-2), and small hydrophobic (SH) proteins have on the pathways of IFN response induction in the infected cells.

### 4.1. Attachment (G) Protein

One of the HMPV proteins that regulates the IFN response in HMPV infection, is the attachment (G) protein. G protein is a type II transmembrane glycoprotein. Albeit the fact that the postulated functional role of G protein is for attachment to a host cell receptor, it is not essential for viral replication [116]. That has been demonstrated in studies where the replication of the mutant virus lacking G protein (rHMPVΔG) in challenged nonhuman primates was strongly attenuated compared to its wild-type counterpart (rHMPV) [9,116], demonstrating that G protein is dispensable for HMPV replication, but those findings also suggested that G protein interferes with the antiviral response. In fact, the G protein is implicated to have an inhibitory effect on RIG-I mediated activation of type I IFN (IFN-α/β) production in airway epithelial cells [89] and in human moDC [105]. Furthermore, our laboratory has shown that the HMPV G protein also attenuates the activation and production of IFN-λ in vitro and in vivo [57] and that the release of IFN-α is also compromised in the airways of infected mice, by the effect of the G protein [90]. The protective immunogenicity properties exhibited by the rHMPVΔG virus makes it a potential vaccine candidate.

### 4.2. Matrix 2 (M2) Protein

Another HMPV protein that has been studied extensively is the second matrix (M2) protein. The HMPV M2 gene contains two overlapping open reading frames (ORF) that encodes two putative proteins: M2-1 and M2-2 (Figure 1). The HMPV M2-1 protein is dispensable for viral recovery in in vitro studies [117], compared to its RSV counterpart, which is important for the transcription of the viral genome in vitro [118]. However, the expression of M2-1 appears to be necessary for HMPV replication in vivo [117]. The complexity of the in vivo environment might account for the discrepancy of HMPV replication in the in vitro experiments when using the M2-1 mutant virus. As for M2-2 protein, the replication of M2-2 mutant HMPV was also highly attenuated in challenged hamsters and African green monkeys, and at the same time confers high levels of immunogenicity and protective efficacy [9,117,119], thus making it a favorable vaccine candidate that needs further testing. Other studies have shown that the M2-2 viral protein suppresses the host innate immunity. For instance, infection of human airway cells with rHMPVΔM2-2 resulted in a significant increase in IFN-β secretion, compared to infection with its wild type counterpart [111]. This outcome is suggested to be as a result of the M2-2 protein targeting the mitochondrial antiviral-signaling protein (MAVS) leading to the blockage of IFN-β transcription [111,112]. Additional data from the same group demonstrated that deletion of the M2-2 ORF protein leads to higher IFN-α, IFN-β, and IFN-γ secretion by human monocyte-derived dendritic cells (moDCs) [120]. They illustrated that the M2-2 protein targets the MyD88 mediated signaling pathway. In contrast to this work, Kitagawa et al. show that the M2-2 protein does not have an inhibitory effect on MyD88-induced NF-κB activation. However, they confirmed that removal of the M2-2 protein upregulates the production of IFN-α by human plasmacytoid dendritic cells (pDCs) via TLR7/9-dependent pathway [113] (Figure 3).

### 4.3. Small Hydrophobic (SH) Protein

Like G protein, the SH protein is a type II membrane protein. The function of the SH protein is not fully known. It is suggested that the SH protein of the HMPV act as a viroporin increasing cell fusion and permeability, similar to RSV [13,121]. Reports using the recombinant HMPV virus lacking the SH protein (rHMPVΔSH), indicate that the mutated virus was capable of growing comparably to the wild type HMPV, as demonstrated in samples from the upper and lower respiratory tract of hamsters. However, the mutated virus (rHMPVΔSH) replicated slightly less well in non-human primates [9,116]. In an in vitro model of infection with rHMPVΔSH, there is also an indication that the protein is not essential for viral replication [122]. Experimental evidence demonstrates that the SH protein of HMPV inhibits type I IFN signaling pathway by blocking the phosphorylation of signal transducer and activator of transcription-1 (STAT1) [114]. A recent study also shows that HMPV SH protein inhibits the production type I IFN in purified human pDCs after HMPV infection [115]. In addition, it has been observed that HMPV uses the TLR7/MyD88 pathway to induce type I IFN response in murine pDCs and the pathway is targeted by the SH protein [115]. Thus far, there are very few reports depicting the role of the HMPV SH protein in regulating the IFN response. Therefore, it is imperative that further work is done to fully understand the function of SH protein on the antiviral response to HMPV infection (Figure 3).

## 5. Conclusions

Although discovered only 17 years ago, the study of the HMPV infection by several groups around the globe has yielded substantial information that furthers our understanding of the complex interplay between HMPV and the IFN response. However, there are still many gaps of knowledge regarding the regulation of the antiviral response to HMPV that need to be defined, such as the role of other less known subtypes of IFN in HMPV infection, the effect of IFN-induced proteins on HMPV replication, the effect of age on the IFN induction, and the contribution of HMPV-induced IFNs on the adaptive immunity.

## Figures and Tables

**Table 1:** Type of IFNs induced by different HMPV strains.

(Group) Virus Strain	IFN Induced	System	Species	Sample (Cells)	References
(A) CAN97-83	IFN-α, β, λ	In vitro	Human	A549, moDC	[57,87,88,96]
(A) CAN97-83	IFN-α, β, λ, γ	In vivo	Mouse	BAL	[57,91,92]
(A) CAN97-83	IFN-α, β	Ex vivo	Mouse	pDC, cDC	[97]
(A) NL1100 (B) NL199	IFN-α, β	In vitro	Human	Monocytes	[86]
(A) D03-574	IFN-γ	In vivo	Mouse	BAL	[93]

moDC = monocyte-derived dendritic cells; pDC = plasmacytoid dendritic cells; cDC = conventional dendritic cells; BAL = bronchoalveolar lavage.

**Table 2:** HMPV proteins that target cellular molecules to inhibit IFN response.

(Group) Virus Strain	HMPV Protein	Cellular Target	References
(A) CAN97-83	G	RIG-I, MDA-5, TLR4	[57,89,105]
(A) CAN97-83	M2-2	MAVS,	[111,112]
(A) Jpn03-1	M2-2	TLR7/9, MyD88	[113]
(A) TN/94-49	SH	STAT1	[114]
(A) CAN97-83	SH	TLR7, MyD88	[115]

## References

[1] Amarasinghe G.K., Bao Y., Basler C.F., Bavari S., Beer M., Bejerman N., Blasdell K.R., Bochnowski A., Briese T., Bukreyev A. (2017). Taxonomy of the order Mononegavirales: Update 2017. Arch. Virol..

[2] Afonso C.L., Amarasinghe G.K., Banyai K., Bao Y., Basler C.F., Bavari S., Bejerman N., Blasdell K.R., Briand F.X., Briese T. (2016). Taxonomy of the order Mononegavirales: Update 2016. Arch. Virol..

[3] Amarasinghe G.K., Arechiga Ceballos N.G., Banyard A.C., Basler C.F., Bavari S., Bennett A.J., Blasdell K.R., Briese T., Bukreyev A., Cai Y. (2018). Taxonomy of the order Mononegavirales: Update 2018. Arch. Virol..

[4] Van den Hoogen B.G., Bestebroer T.M., Osterhaus A.D., Fouchier R.A. (2002). Analysis of the genomic sequence of a human metapneumovirus. Virology.

[5] Cheemarla N.R., Guerrero-Plata A. (2015). Immune Response to Human Metapneumovirus Infection: What We Have Learned from the Mouse Model. Pathogens.

[6] Collins P.L., Crowe J., Knipe D.M., Howley P.M. (2007). Respiratory Syncytial Virus and Metapneumovirus. Fileds Virology.

[7] Cox R.G., Livesay S.B., Johnson M., Ohi M.D., Williams J.V. (2012). The human metapneumovirus fusion protein mediates entry via an interaction with RGD-binding integrins. J. Virol..

[8] Biacchesi S., Skiadopoulos M.H., Boivin G., Hanson C.T., Murphy B.R., Collins P.L., Buchholz U.J. (2003). Genetic diversity between human metapneumovirus subgroups. Virology.

[9] Biacchesi S., Pham Q.N., Skiadopoulos M.H., Murphy B.R., Collins P.L., Buchholz U.J. (2005). Infection of nonhuman primates with recombinant human metapneumovirus lacking the SH, G., or M2-2 protein categorizes each as a nonessential accessory protein and identifies vaccine candidates. J. Virol..

[10] Mackay I.M., Jacob K.C., Woolhouse D., Waller K., Syrmis M.W., Whiley D.M., Siebert D.J., Nissen M., Sloots T.P. (2003). Molecular assays for detection of human metapneumovirus. J. Clin. Microbiol..

[11] Easton A.J., Domachowske J.B., Rosenberg H.F. (2004). Animal pneumoviruses: Molecular genetics and pathogenesis. Clin. Microbiol. Rev..

[12] Cai H., Zhang Y., Lu M., Liang X., Jennings R., Niewiesk S., Li J. (2016). Phosphorylation of Human Metapneumovirus M2-1 Protein Upregulates Viral Replication and Pathogenesis. J. Virol..

[13] Masante C., El Najjar F., Chang A., Jones A., Moncman C.L., Dutch R.E. (2014). The human metapneumovirus small hydrophobic protein has properties consistent with those of a viroporin and can modulate viral fusogenic activity. J. Virol..

[14] Van den Hoogen B.G., Herfst S., Sprong L., Cane P.A., Forleo-Neto E., de Swart R.L., Osterhaus A.D., Fouchier R.A. (2004). Antigenic and genetic variability of human metapneumoviruses. Emerg. Infect. Dis..

[15] Bastien N., Normand S., Taylor T., Ward D., Peret T.C., Boivin G., Anderson L.J., Li Y. (2003). Sequence analysis of the N., P., *M* and *F* genes of Canadian human metapneumovirus strains. Virus Res..

[16] Fearns R., Collins P.L. (1999). Model for polymerase access to the overlapped L gene of respiratory syncytial virus. J. Virol..

[17] Van den Hoogen B.G., de Jong J.C., Groen J., Kuiken T., de Groot R., Fouchier R.A., Osterhaus A.D. (2001). A newly discovered human pneumovirus isolated from young children with respiratory tract disease. Nat. Med..

[18] Williams J.V., Harris P.A., Tollefson S.J., Halburnt-Rush L.L., Pingsterhaus J.M., Edwards K.M., Wright P.F., Crowe J.E. (2004). Human metapneumovirus and lower respiratory tract disease in otherwise healthy infants and children. N. Engl. J. Med..

[19] Kahn J.S. (2006). Epidemiology of human metapneumovirus. Clin. Microbiol. Rev..

[20] Van den Hoogen B.G., van Doornum G.J., Fockens J.C., Cornelissen J.J., Beyer W.E., de Groot R., Osterhaus A.D., Fouchier R.A. (2003). Prevalence and clinical symptoms of human metapneumovirus infection in hospitalized patients. J. Infect. Dis..

[21] Samuel S., Nanjappa S., Cooper C.D., Greene J.N. (2016). Human Metapneumovirus Infection in Immunocompromised Patients. Cancer Control.

[22] Mullins J.A., Erdman D.D., Weinberg G.A., Edwards K., Hall C.B., Walker F.J., Iwane M., Anderson L.J. (2004). Human metapneumovirus infection among children hospitalized with acute respiratory illness. Emerg. Infect. Dis..

[23] Haas L.E., Thijsen S.F., van Elden L., Heemstra K.A. (2013). Human metapneumovirus in adults. Viruses.

[24] Boivin G., De Serres G., Cote S., Gilca R., Abed Y., Rochette L., Bergeron M.G., Dery P. (2003). Human metapneumovirus infections in hospitalized children. Emerg. Infect. Dis..

[25] Caracciolo S., Minini C., Colombrita D., Rossi D., Miglietti N., Vettore E., Caruso A., Fiorentini S. (2008). Human metapneumovirus infection in young children hospitalized with acute respiratory tract disease: Virologic and clinical features. Pediatr. Infect. Dis. J..

[26] Williams J.V., Wang C.K., Yang C.F., Tollefson S.J., House F.S., Heck J.M., Chu M., Brown J.B., Lintao L.D., Quinto J.D. (2006). The role of human metapneumovirus in upper respiratory tract infections in children: A 20-year experience. J. Infect. Dis..

[27] Boivin G., de Serres G., Hamelin M.E., Cote S., Argouin M., Tremblay G., Maranda-Aubut R., Sauvageau C., Ouakki M., Boulianne N. (2007). An outbreak of severe respiratory tract infection due to human metapneumovirus in a long-term care facility. Clin. Infect. Dis..

[28] Englund J.A., Boeckh M., Kuypers J., Nichols W.G., Hackman R.C., Morrow R.A., Fredricks D.N., Corey L. (2006). Brief communication: Fatal human metapneumovirus infection in stem-cell transplant recipients. Ann. Intern. Med..

[29] Rohde G., Borg I., Arinir U., Kronsbein J., Rausse R., Bauer T.T., Bufe A., Schultze-Werninghaus G. (2005). Relevance of human metapneumovirus in exacerbations of COPD. Respir. Res..

[30] Tollefson S.J., Cox R.G., Williams J.V. (2010). Studies of culture conditions and environmental stability of human metapneumovirus. Virus Res..

[31] Matsuzaki Y., Itagaki T., Ikeda T., Aoki Y., Abiko C., Mizuta K. (2013). Human metapneumovirus infection among family members. Epidemiol. Infect..

[32] Tu C.C., Chen L.K., Lee Y.S., Ko C.F., Chen C.M., Yang H.H., Lee J.J. (2009). An outbreak of human metapneumovirus infection in hospitalized psychiatric adult patients in Taiwan. Scand. J. Infect. Dis..

[33] Lessler J., Reich N.G., Brookmeyer R., Perl T.M., Nelson K.E., Cummings D.A. (2009). Incubation periods of acute respiratory viral infections: A systematic review. Lancet Infect. Dis..

[34] Peiris J.S., Tang W.H., Chan K.H., Khong P.L., Guan Y., Lau Y.L., Chiu S.S. (2003). Children with respiratory disease associated with metapneumovirus in Hong Kong. Emerg. Infect. Dis..

[35] Jartti T., Lehtinen P., Vuorinen T., Osterback R., van den Hoogen B., Osterhaus A.D., Ruuskanen O. (2004). Respiratory picornaviruses and respiratory syncytial virus as causative agents of acute expiratory wheezing in children. Emerg. Infect. Dis..

[36] Boivin G., Abed Y., Pelletier G., Ruel L., Moisan D., Cote S., Peret T.C., Erdman D.D., Anderson L.J. (2002). Virological features and clinical manifestations associated with human metapneumovirus: A new paramyxovirus responsible for acute respiratory-tract infections in all age groups. J. Infect. Dis..

[37] Schuster J.E., Khuri-Bulos N., Faouri S., Shehabi A., Johnson M., Wang L., Fonnesbeck C., Williams J.V., Halasa N. (2015). Human Metapneumovirus Infection in Jordanian Children: Epidemiology and Risk Factors for Severe Disease. Pediatr. Infect. Dis. J..

[38] Edwards K.M., Zhu Y., Griffin M.R., Weinberg G.A., Hall C.B., Szilagyi P.G., Staat M.A., Iwane M., Prill M.M., Williams J.V. (2013). Burden of human metapneumovirus infection in young children. N. Engl. J. Med..

[39] Papenburg J., Hamelin M.E., Ouhoummane N., Carbonneau J., Ouakki M., Raymond F., Robitaille L., Corbeil J., Caouette G., Frenette L. (2012). Comparison of risk factors for human metapneumovirus and respiratory syncytial virus disease severity in young children. J. Infect. Dis..

[40] Anderson E.J., Simoes E.A., Buttery J.P., Dennehy P.H., Domachowske J.B., Jensen K., Lieberman J.M., Losonsky G.A., Yogev R. (2012). Prevalence and Characteristics of Human Metapneumovirus Infection Among Hospitalized Children at High Risk for Severe Lower Respiratory Tract Infection. J. Pediatr. Infect. Dis. Soc..

[41] Pancham K., Sami I., Perez G.F., Huseni S., Kurdi B., Rose M.C., Rodriguez-Martinez C.E., Nino G. (2016). Human Metapneumovirus Infection is Associated with Severe Respiratory Disease in Preschool Children with History of Prematurity. Pediatr. Neonatol..

[42] Hahn A., Wang W., Jaggi P., Dvorchik I., Ramilo O., Koranyi K., Mejias A. (2013). Human metapneumovirus infections are associated with severe morbidity in hospitalized children of all ages. Epidemiol. Infect..

[43] Spaeder M.C., Custer J.W., Bembea M.M., Aganga D.O., Song X., Scafidi S. (2013). A multicenter outcomes analysis of children with severe viral respiratory infection due to human metapneumovirus. Pediatr. Crit. Care Med..

[44] Moe N., Krokstad S., Stenseng I.H., Christensen A., Skanke L.H., Risnes K.R., Nordbo S.A., Dollner H. (2017). Comparing Human Metapneumovirus and Respiratory Syncytial Virus: Viral Co-Detections, Genotypes and Risk Factors for Severe Disease. PLoS ONE.

[45] Kitanovski L., Kopriva S., Pokorn M., Dolnicar M.B., Rajic V., Stefanovic M., Jazbec J. (2013). Treatment of severe human metapneumovirus (hMPV) pneumonia in an immunocompromised child with oral ribavirin and IVIG. J. Pediatr. Hematol. Oncol..

[46] Deffrasnes C., Hamelin M.E., Prince G.A., Boivin G. (2008). Identification and evaluation of a highly effective fusion inhibitor for human metapneumovirus. Antimicrob. Agents Chemother..

[47] Deffrasnes C., Cavanagh M.H., Goyette N., Cui K., Ge Q., Seth S., Templin M.V., Quay S.C., Johnson P.H., Boivin G. (2008). Inhibition of human metapneumovirus replication by small interfering RNA. Antivir. Ther..

[48] McNab F., Mayer-Barber K., Sher A., Wack A., O’Garra A. (2015). Type I interferons in infectious disease. Nat. Rev. Immunol..

[49] Van Pesch V., Lanaya H., Renauld J.C., Michiels T. (2004). Characterization of the murine alpha interferon gene family. J. Virol..

[50] Pestka S., Krause C.D., Walter M.R. (2004). Interferons, interferon-like cytokines, and their receptors. Immunol. Rev..

[51] Gibbert K., Schlaak J.F., Yang D., Dittmer U. (2013). IFN-alpha subtypes: Distinct biological activities in anti-viral therapy. Br. J. Pharmacol..

[52] Colonna M., Trinchieri G., Liu Y.J. (2004). Plasmacytoid dendritic cells in immunity. Nat. Immunol..

[53] Schroder K., Hertzog P.J., Ravasi T., Hume D.A. (2004). Interferon-gamma: An overview of signals, mechanisms and functions. J. Leukoc. Biol..

[54] Donnelly R.P., Kotenko S.V. (2010). Interferon-lambda: A new addition to an old family. J. Interf. Cytokine Res..

[55] Kotenko S.V., Durbin J.E. (2017). Contribution of type III interferons to antiviral immunity: Location, location, location. J. Biol. Chem..

[56] Yin Z., Dai J., Deng J., Sheikh F., Natalia M., Shih T., Lewis-Antes A., Amrute S.B., Garrigues U., Doyle S. (2012). Type III IFNs are produced by and stimulate human plasmacytoid dendritic cells. J. Immunol..

[57] Banos-Lara Mdel R., Harvey L., Mendoza A., Simms D., Chouljenko V.N., Wakamatsu N., Kousoulas K.G., Guerrero-Plata A. (2015). Impact and regulation of lambda interferon response in human metapneumovirus infection. J. Virol..

[58] Takeda K., Akira S. (2005). Toll-like receptors in innate immunity. Int. Immunol..

[59] Thompson A.J., Locarnini S.A. (2007). Toll-like receptors, RIG-I-like RNA helicases and the antiviral innate immune response. Immunol. Cell Biol..

[60] Kato H., Takeuchi O., Sato S., Yoneyama M., Yamamoto M., Matsui K., Uematsu S., Jung A., Kawai T., Ishii K.J. (2006). Differential roles of MDA5 and RIG-I helicases in the recognition of RNA viruses. Nature.

[61] Loo Y.M., Fornek J., Crochet N., Bajwa G., Perwitasari O., Martinez-Sobrido L., Akira S., Gill M.A., Garcia-Sastre A., Katze M.G. (2008). Distinct RIG-I and MDA5 signaling by RNA viruses in innate immunity. J. Virol..

[62] Hornung V., Ellegast J., Kim S., Brzozka K., Jung A., Kato H., Poeck H., Akira S., Conzelmann K.K., Schlee M. (2006). 5′-Triphosphate RNA is the ligand for RIG-I. Science.

[63] Pichlmair A., Schulz O., Tan C.P., Naslund T.I., Liljestrom P., Weber F., Reis e Sousa C. (2006). RIG-I-mediated antiviral responses to single-stranded RNA bearing 5′-phosphates. Science.

[64] Kato H., Takeuchi O., Mikamo-Satoh E., Hirai R., Kawai T., Matsushita K., Hiiragi A., Dermody T.S., Fujita T., Akira S. (2008). Length-dependent recognition of double-stranded ribonucleic acids by retinoic acid-inducible gene-I and melanoma differentiation-associated gene 5. J. Exp. Med..

[65] Sedger L.M. (2013). microRNA control of interferons and interferon induced anti-viral activity. Mol. Immunol..

[66] Lee R.C., Feinbaum R.L., Ambros V. (1993). The *C. elegans* heterochronic gene lin-4 encodes small RNAs with antisense complementarity to lin-14. Cell.

[67] Melamed Z., Levy A., Ashwal-Fluss R., Lev-Maor G., Mekahel K., Atias N., Gilad S., Sharan R., Levy C., Kadener S. (2013). Alternative splicing regulates biogenesis of miRNAs located across exon-intron junctions. Mol. Cell.

[68] Krol J., Loedige I., Filipowicz W. (2010). The widespread regulation of microRNA biogenesis, function and decay. Nat. Rev. Genet..

[69] Nadeem A., Ashraf M.R., Javed M., Hussain T., Tariq M.S., Babar M.E. (2018). Review-MicroRNAs: A new paradigm towards mechanistic insight of diseases. Pak. J. Pharm. Sci..

[70] He L., Hannon G.J. (2004). MicroRNAs: Small RNAs with a big role in gene regulation. Nat. Rev. Genet..

[71] Sato T., Baskoro H., Rennard S.I., Seyama K., Takahashi K. (2015). MicroRNAs as Therapeutic Targets in Lung Disease: Prospects and Challenges. Chronic Obstr. Pulm. Dis..

[72] Polioudakis D., Bhinge A.A., Killion P.J., Lee B.K., Abell N.S., Iyer V.R. (2013). A Myc-microRNA network promotes exit from quiescence by suppressing the interferon response and cell-cycle arrest genes. Nucleic Acids Res..

[73] Su C., Hou Z., Zhang C., Tian Z., Zhang J. (2011). Ectopic expression of microRNA-155 enhances innate antiviral immunity against HBV infection in human hepatoma cells. Virol. J..

[74] Banos-Lara M.D.R., Zabaleta J., Garai J., Baddoo M., Guerrero-Plata A. (2018). Comparative analysis of miRNA profile in human dendritic cells infected with respiratory syncytial virus and human metapneumovirus. BMC Res. Notes.

[75] Deng J., Ptashkin R.N., Wang Q., Liu G., Zhang G., Lee I., Lee Y.S., Bao X. (2014). Human metapneumovirus infection induces significant changes in small noncoding RNA expression in airway epithelial cells. Mol. Ther. Nucleic Acids.

[76] Rupani H., Sanchez-Elsner T., Howarth P. (2013). MicroRNAs and respiratory diseases. Eur. Respir. J..

[77] Metz P., Dazert E., Ruggieri A., Mazur J., Kaderali L., Kaul A., Zeuge U., Windisch M.P., Trippler M., Lohmann V. (2012). Identification of type I and type II interferon-induced effectors controlling hepatitis C. virus replication. Hepatology.

[78] Zhang Y., Burke C.W., Ryman K.D., Klimstra W.B. (2007). Identification and characterization of interferon-induced proteins that inhibit alphavirus replication. J. Virol..

[79] Schoggins J.W., Wilson S.J., Panis M., Murphy M.Y., Jones C.T., Bieniasz P., Rice C.M. (2011). A diverse range of gene products are effectors of the type I interferon antiviral response. Nature.

[80] Schoggins J.W., MacDuff D.A., Imanaka N., Gainey M.D., Shrestha B., Eitson J.L., Mar K.B., Richardson R.B., Ratushny A.V., Litvak V. (2014). Pan-viral specificity of IFN-induced genes reveals new roles for cGAS in innate immunity. Nature.

[81] McMichael T.M., Zhang Y., Kenney A.D., Zhang L., Zani A., Lu M., Chemudupati M., Li J., Yount J.S. (2018). IFITM3 Restricts Human Metapneumovirus Infection. J. Infect. Dis..

[82] Zhang W., Zhang L., Zan Y., Du N., Yang Y., Tien P. (2015). Human respiratory syncytial virus infection is inhibited by IFN-induced transmembrane proteins. J. Gen. Virol..

[83] Everitt A.R., Clare S., McDonald J.U., Kane L., Harcourt K., Ahras M., Lall A., Hale C., Rodgers A., Young D.B. (2013). Defining the range of pathogens susceptible to Ifitm3 restriction using a knockout mouse model. PLoS ONE.

[84] Jumat M.R., Huong T.N., Ravi L.I., Stanford R., Tan B.H., Sugrue R.J. (2015). Viperin protein expression inhibits the late stage of respiratory syncytial virus morphogenesis. Antivir. Res..

[85] Gonzalez-Sanz R., Mata M., Bermejo-Martin J., Alvarez A., Cortijo J., Melero J.A., Martinez I. (2016). ISG15 Is Upregulated in Respiratory Syncytial Virus Infection and Reduces Virus Growth through Protein ISGylation. J. Virol..

[86] Goutagny N., Jiang Z., Tian J., Parroche P., Schickli J., Monks B.G., Ulbrandt N., Ji H., Kiener P.A., Coyle A.J. (2010). Cell type-specific recognition of human metapneumoviruses (HMPVs) by retinoic acid-inducible gene I (RIG-I) and TLR7 and viral interference of RIG-I ligand recognition by HMPV-B1 phosphoprotein. J. Immunol..

[87] Liao S., Bao X., Liu T., Lai S., Li K., Garofalo R.P., Casola A. (2008). Role of retinoic acid inducible gene-I in human metapneumovirus-induced cellular signalling. J. Gen. Virol..

[88] Banos-Lara Mdel R., Ghosh A., Guerrero-Plata A. (2013). Critical role of MDA5 in the interferon response induced by human metapneumovirus infection in dendritic cells and in vivo. J. Virol..

[89] Bao X., Liu T., Shan Y., Li K., Garofalo R.P., Casola A. (2008). Human metapneumovirus glycoprotein G inhibits innate immune responses. PLoS Pathog..

[90] Cheemarla N.R., Guerrero-Plata A. (2017). Human Metapneumovirus Attachment Protein Contributes to Neutrophil Recruitment into the Airways of Infected Mice. Viruses.

[91] Guerrero-Plata A., Baron S., Poast J.S., Adegboyega P.A., Casola A., Garofalo R.P. (2005). Activity and regulation of alpha interferon in respiratory syncytial virus and human metapneumovirus experimental infections. J. Virol..

[92] Guerrero-Plata A., Casola A., Garofalo R.P. (2005). Human metapneumovirus induces a profile of lung cytokines distinct from that of respiratory syncytial virus. J. Virol..

[93] Huck B., Neumann-Haefelin D., Schmitt-Graeff A., Weckmann M., Mattes J., Ehl S., Falcone V. (2007). Human metapneumovirus induces more severe disease and stronger innate immune response in BALB/c mice as compared with respiratory syncytial virus. Respir. Res..

[94] Le Nouen C., Munir S., Losq S., Winter C.C., McCarty T., Stephany D.A., Holmes K.L., Bukreyev A., Rabin R.L., Collins P.L. (2009). Infection and maturation of monocyte-derived human dendritic cells by human respiratory syncytial virus, human metapneumovirus, and human parainfluenza virus type 3. Virology.

[95] Guerrero-Plata A. (2013). Dendritic cells in human Pneumovirus and Metapneumovirus infections. Viruses.

[96] Bao X., Liu T., Spetch L., Kolli D., Garofalo R.P., Casola A. (2007). Airway epithelial cell response to human metapneumovirus infection. Virology.

[97] Guerrero-Plata A., Kolli D., Hong C., Casola A., Garofalo R.P. (2009). Subversion of pulmonary dendritic cell function by paramyxovirus infections. J. Immunol..

[98] Malmo J., Moe N., Krokstad S., Ryan L., Loevenich S., Johnsen I.B., Espevik T., Nordbo S.A., Dollner H., Anthonsen M.W. (2016). Cytokine Profiles in Human Metapneumovirus Infected Children: Identification of Genes Involved in the Antiviral Response and Pathogenesis. PLoS ONE.

[99] Melendi G.A., Laham F.R., Monsalvo A.C., Casellas J.M., Israele V., Polack N.R., Kleeberger S.R., Polack F.P. (2007). Cytokine profiles in the respiratory tract during primary infection with human metapneumovirus, respiratory syncytial virus, or influenza virus in infants. Pediatrics.

[100] Pancham K., Perez G.F., Huseni S., Jain A., Kurdi B., Rodriguez-Martinez C.E., Preciado D., Rose M.C., Nino G. (2015). Premature infants have impaired airway antiviral IFNgamma responses to human metapneumovirus compared to respiratory syncytial virus. Pediatr. Res..

[101] Biacchesi S., Pham Q.N., Skiadopoulos M.H., Murphy B.R., Collins P.L., Buchholz U.J. (2006). Modification of the trypsin-dependent cleavage activation site of the human metapneumovirus fusion protein to be trypsin independent does not increase replication or spread in rodents or nonhuman primates. J. Virol..

[102] Dinwiddie D.L., Harrod K.S. (2008). Human metapneumovirus inhibits IFN-alpha signaling through inhibition of STAT1 phosphorylation. Am. J. Respir. Cell Mol. Biol..

[103] Rinaldo C.R., Piazza P. (2004). Virus infection of dendritic cells: Portal for host invasion and host defense. Trends Microbiol..

[104] Guerrero-Plata A., Casola A., Suarez G., Yu X., Spetch L., Peeples M.E., Garofalo R.P. (2006). Differential response of dendritic cells to human metapneumovirus and respiratory syncytial virus. Am. J. Respir. Cell Mol. Biol..

[105] Kolli D., Bao X., Liu T., Hong C., Wang T., Garofalo R.P., Casola A. (2011). Human metapneumovirus glycoprotein G inhibits TLR4-dependent signaling in monocyte-derived dendritic cells. J. Immunol..

[106] Douville R.N., Bastien N., Li Y., Pochard P., Simons F.E., HayGlass K.T. (2006). Human metapneumovirus elicits weak IFN-gamma memory responses compared with respiratory syncytial virus. J. Immunol..

[107] Zhang W., Yang H., Kong X., Mohapatra S., San Juan-Vergara H., Hellermann G., Behera S., Singam R., Lockey R.F., Mohapatra S.S. (2005). Inhibition of respiratory syncytial virus infection with intranasal siRNA nanoparticles targeting the viral NS1 gene. Nat. Med..

[108] Schlender J., Bossert B., Buchholz U., Conzelmann K.K. (2000). Bovine respiratory syncytial virus nonstructural proteins NS1 and NS2 cooperatively antagonize alpha/beta interferon-induced antiviral response. J. Virol..

[109] Hastings A.K., Erickson J.J., Schuster J.E., Boyd K.L., Tollefson S.J., Johnson M., Gilchuk P., Joyce S., Williams J.V. (2015). Role of type I interferon signaling in human metapneumovirus pathogenesis and control of viral replication. J. Virol..

[110] Darniot M., Pitoiset C., Petrella T., Aho S., Pothier P., Manoha C. (2009). Age-associated aggravation of clinical disease after primary metapneumovirus infection of BALB/C mice. J. Virol..

[111] Ren J., Wang Q., Kolli D., Prusak D.J., Tseng C.T., Chen Z.J., Li K., Wood T.G., Bao X. (2012). Human metapneumovirus M2-2 protein inhibits innate cellular signaling by targeting MAVS. J. Virol..

[112] Chen Y., Deng X., Deng J., Zhou J., Ren Y., Liu S., Prusak D.J., Wood T.G., Bao X. (2016). Functional motifs responsible for human metapneumovirus M2-2-mediated innate immune evasion. Virology.

[113] Kitagawa Y., Sakai M., Funayama M., Itoh M., Gotoh B. (2017). Human Metapneumovirus M2-2 Protein Acts as a Negative Regulator of Alpha Interferon Production by Plasmacytoid Dendritic Cells. J. Virol..

[114] Hastings A.K., Amato K.R., Wen S.C., Peterson L.S., Williams J.V. (2016). Human metapneumovirus small hydrophobic (SH) protein downregulates type I IFN pathway signaling by affecting STAT1 expression and phosphorylation. Virology.

[115] Bao X., Kolli D., Esham D., Velayutham T.S., Casola A. (2018). Human Metapneumovirus Small Hydrophobic Protein Inhibits Interferon Induction in Plasmacytoid Dendritic Cells. Viruses.

[116] Biacchesi S., Skiadopoulos M.H., Yang L., Lamirande E.W., Tran K.C., Murphy B.R., Collins P.L., Buchholz U.J. (2004). Recombinant human Metapneumovirus lacking the small hydrophobic SH and/or attachment G glycoprotein: Deletion of G yields a promising vaccine candidate. J. Virol..

[117] Buchholz U.J., Biacchesi S., Pham Q.N., Tran K.C., Yang L., Luongo C.L., Skiadopoulos M.H., Murphy B.R., Collins P.L. (2005). Deletion of M2 gene open reading frames 1 and 2 of human metapneumovirus: Effects on RNA synthesis, attenuation, and immunogenicity. J. Virol..

[118] Sutherland K.A., Collins P.L., Peeples M.E. (2001). Synergistic effects of gene-end signal mutations and the M2-1 protein on transcription termination by respiratory syncytial virus. Virology.

[119] Schickli J.H., Kaur J., Macphail M., Guzzetta J.M., Spaete R.R., Tang R.S. (2008). Deletion of human metapneumovirus M2-2 increases mutation frequency and attenuates growth in hamsters. Virol. J..

[120] Ren J., Liu G., Go J., Kolli D., Zhang G., Bao X. (2014). Human metapneumovirus M2-2 protein inhibits innate immune response in monocyte-derived dendritic cells. PLoS ONE.

[121] Gan S.W., Tan E., Lin X., Yu D., Wang J., Tan G.M., Vararattanavech A., Yeo C.Y., Soon C.H., Soong T.W. (2012). The small hydrophobic protein of the human respiratory syncytial virus forms pentameric ion channels. J. Biol. Chem..

[122] De Graaf M., Herfst S., Aarbiou J., Burgers P.C., Zaaraoui-Boutahar F., Bijl M., van Ijcken W., Schrauwen E.J., Osterhaus A.D., Luider T.M. (2013). Small hydrophobic protein of human metapneumovirus does not affect virus replication and host gene expression in vitro. PLoS ONE.

